# Profile of orthopaedic day-case procedures at a district general hospital (retrospective study)

**DOI:** 10.1186/s13018-023-04366-4

**Published:** 2023-11-20

**Authors:** Mansour Mohammed Aldhilan, Azeez Olalekan Tella

**Affiliations:** 1 Alrass General Hospital, Ministry of Health, Alrass, Saudi Arabia; 2 Department Orthopaedics, Samtah General Hospital, Ministry of Health, Samtah, Saudi Arabia

**Keywords:** Orthopaedics, Day-case procedure, District

## Abstract

**Background:**

The outcomes of orthopaedic day-case procedures have been reported widely, but there is a lack of reports from secondary health facilities such as district hospitals.

**Aim:**

We aimed to perform a retrospective analysis of patient records to capture the profile of day-case procedures performed.

**Materials and methods:**

We conducted a retrospective analysis of day-case procedures at the dedicated Day Surgery Unit of a moderate-sized district hospital in Saudi Arabia between January 2021 and December 2022. The medical records of all the patients who had day-case procedures by the hospital's orthopaedic unit were analyzed.

**Results:**

Within the study period, 71 out of 914 elective orthopaedic procedures were carried out as day-cases, giving a day-case surgery rate of 7.8%. The mean age was 25.3 ± 12.2 (range, 4–55 years), and the male-to-female ratio was 6:1. The spectrum of the procedures was dominated by implant removal in 59 cases (83.1%). Whilst the anaesthetic technique varied, all the patients were ASA class I or II. There were minor complications in 10 patients (14.0%), with 7 of them (9.8%) needing inpatient admission. There was no cancellation of cases in our study.

**Conclusion:**

We found day-case procedures to be safe and effective but with low utilisation of the Day Surgery Unit, which can be improved through the development of a detailed protocol for day surgery in the hospital.

## Introduction

Day-case surgery is the admission of selected patients to the hospital for a planned surgical procedure without an overnight stay in the hospital. This is based on the principle that certain surgical procedures in selected patients can be carried out safely with the patients returning home the same day [1]. In orthopaedic practice, day-case procedures are increasingly being performed due to recognized advantages, including reduced demand for inpatient beds, improved theatre utilisation, cost-effectiveness, and improved patient satisfaction [2, 3]. This invariably leads to a more significant turnover of cases for the individual surgeon and reduced healthcare costs from the hospital’s perspective [4].

Day-case surgeries can also be fraught with disadvantages when the procedures are not adequately planned or when patients are discharged too soon. These include nausea and vomiting, inadequate pain control, the need for unscheduled in-hospital admission and an extra burden being placed on family members and community services [5]. Thus, it is imperative that every aspect of day-case surgery, such as preoperative care, care during surgery, postoperative monitoring and assessment, discharge and postdischarge care, be meticulously planned [5, 6]. The British Association of Day Surgery (BADS), which is a multidisciplinary professional organization, and the International Association for Ambulatory Surgery (IAAS) have helped to develop guidelines and set quality standards regarding day-case surgery over the years [6, 7]. Most hospitals have delivered quality care to these patients by creating a dedicated Day Surgery Unit (DSU) [2].

Orthopaedic day-case procedures continue to gain popularity due to advancements in surgical techniques, anaesthesia, and postoperative care. Limited studies in Saudi Arabia have focused on day-case procedures in General surgery speciality, particularly laparoscopic cholecystectomy in large volume centres [8, 9]. The authors have not found any study focusing on day-case orthopaedic procedures. Our study aims to fill this gap by providing a retrospective analysis of orthopaedic day-case procedures at a district general hospital.

## Materials and methods

After obtaining the hospital’s approval for the study, we conducted a retrospective analysis of patient records to identify orthopaedic day-case procedures performed at the district general hospital between January 2021 and December 2022. The data collected included patient demographics, surgical details (e.g., procedure type, anaesthesia used) and postoperative complications, including conversion to ward admission.

The inclusion criteria included ASA grade I and II patients, procedures with an expected duration of less than 2 h, and patients' informed consent. The exclusion criteria included ASA grade II with uncontrolled comorbid conditions, ASA grade III and above, and patients who live outside the district and do not have relatives within the district where the hospital is located.

Our facility, Samtah General Hospital, is a 150-bed multispecialty district general hospital located in Samtah district, Jazan region (southwestern part of Saudi Arabia). The hospital's Day Surgery Unit (DSU) is a dedicated unit comprising six beds and administered by the nursing staff. The DSU is shared by all surgical specialities (orthopaedics, general surgery, gynaecology, ENT, ophthalmology, and urology) available in the hospital on different days. In orthopaedics, patient selection is usually left at the surgeon’s discretion but follows the inclusion criteria. These include the patient's understanding of the procedure and granting consent for it to be performed as a day-case procedure and the surgeon's assurance that the procedure is not associated with the possibility of major postoperative complications. Other criteria include the patients' ability to contact the hospital by phone should they have any concerns about their procedures.

The selected patients are then referred to the dedicated Anaesthesia Clinic in the hospital, where they are reviewed and, based on the anaesthetist's recommendations, are either accepted or rejected for the procedure to be performed as a day-case. The accepted patients returned to the orthopaedic surgeon to schedule the procedure in conjunction with the staff of the DSU. The patients are then given relevant instructions regarding the fasting period and the timing of their operations. Parents or guardians must accompany children, whilst adults are also instructed to come with responsible adults to accompany them.

The patients report to the DSU on the procedure date as scheduled and are wheeled to the operating room by the DSU staff. After completing their procedures, they are returned to the DSU, where they will be discharged. The discharge criteria generally follow Korttila’s criteria for day surgery: vital signs must be stable for at least one hour; the patient should be oriented in time, place, and person, able to tolerate orally administered fluids, able to void and experience no more than minimal nausea or vomiting. Other criteria include an absence of excessive pain and bleeding, and discharge should be an agreement by both anaesthetist and surgeon [10, 11]. In addition, instructions were given to follow at home, and DSU contact was provided should the patient have concerns. We also ensured explicit consent from the patient or the adult caregiver before discharge, and follow-up appointments at the orthopaedic clinic were given, usually within one week.

Statistical analysis for the study was performed using SPSS (Statistical Package for the Social Sciences) version 26 for Windows. We used descriptive statistics to illustrate data distribution, whilst additional data presentation was done using tables and bar charts.

## Results

A total of 71 patients had day-case procedures during the period under review. The mean age was 25.3 ± 12.2 (range, 4–55 years). The age group distribution revealed that 16 patients (22.5%) were < 16 years (paediatric age group). The sex distribution showed 61 males (85.9%) and ten females (14.1%). During the study period, 914 elective orthopaedic surgeries were carried out, of which 71 were performed as day-case procedures. This gives a day-case rate of 7.8%.

**Fig. 1:**
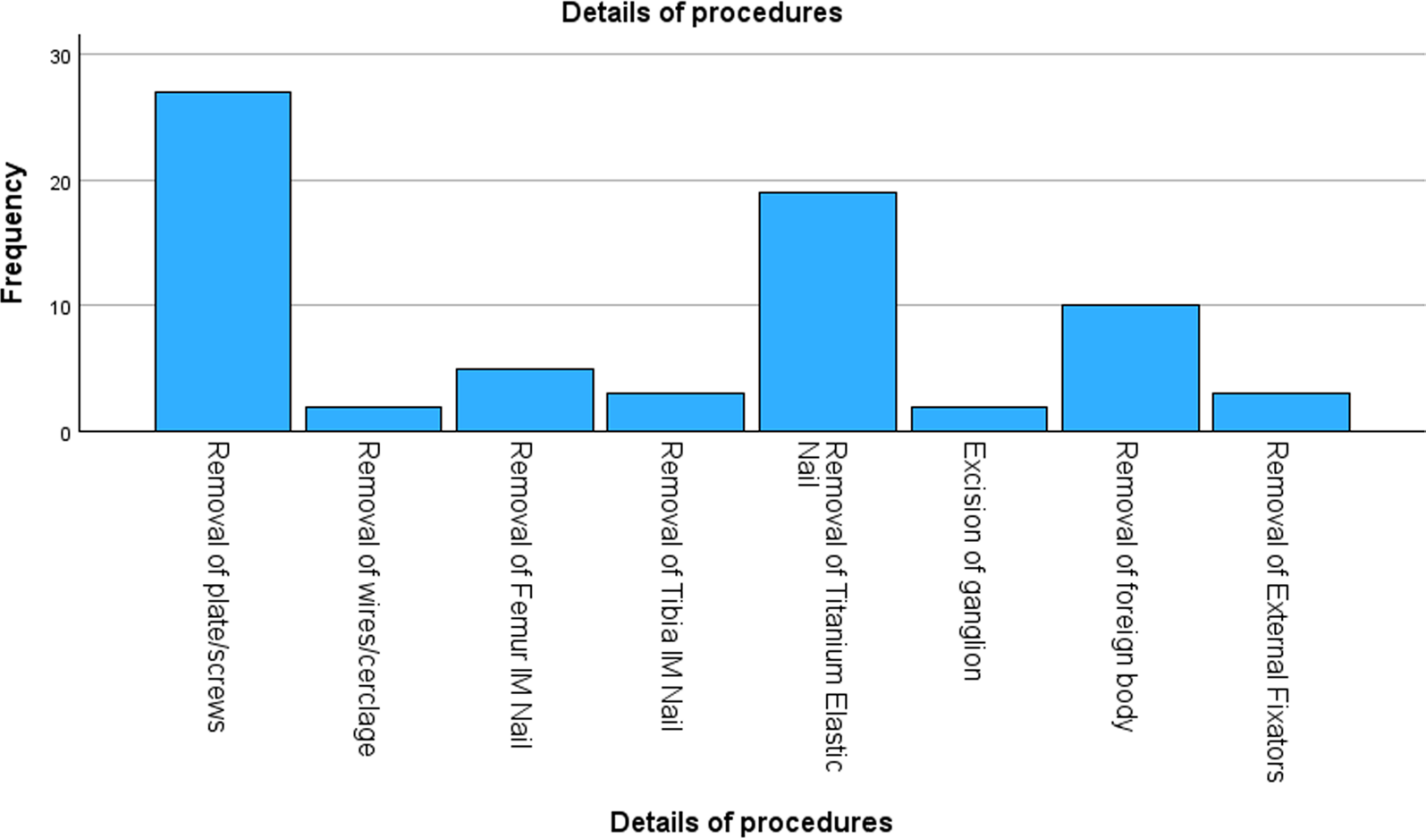
Details of the procedures

The spectrum of procedures carried out revealed the removal of plates and screws as the most common procedure (*n* = 27, 38.0%), followed by removal of titanium elastic nails (*n* = 19, 26.8%) and removal of foreign bodies (*n* = 10, 14.1%). Other procedures were removal of the femoral intramedullary nail (*n* = 5, 7.0%), removal of the tibial intramedullary nail (*n* = 3, 4.2%), rremoval of external fixators (*n* = 3, 4.2%), removal of *k*-wires and cerclage wires from the patella (*n* = 2, 2.8%) and excision of the dorsal wrist ganglion (*n* = 2, 2.8%). The details of the procedures are shown in Fig. 1.

Regarding laterality, 33 procedures (46.5%) were carried out on the right side, whilst the remaining 38 (53.5%) were on the left side. The detailed anatomical site of the surgery is shown in Table 1.

**Table 1:** Details of the anatomical site of surgery

Anatomical site of surgery	Frequency	Percentage
Clavicle	1	1.4
Humerus	9	12.7
Forearm	14	19.7
Wrist	2	2.8
Hand	3	4.2
Femur	20	28.2
Patella	2	2.8
Tibia	5	7.0
Ankle	9	12.7
Foot	6	8.5
Total	71	100.0

The ASA scores were distributed as follows: ASA I in 65 patients (91.5%) and ASA II in 6 patients (8.5%). There were no patients with ASA III and above. Table 2 shows the anaesthesia technique used in the 71 patients.

**Table 2:** Anaesthesia technique used

Anaesthesia technique	Frequency	Percentage of total
General	22	31.0
Local	11	15.5
Spinal	30	42.3
Regional (bier’s block)	8	11.3
Total	71	100.0

Following the completion of the day’s procedures, patients were reviewed by the anaesthetist and the surgical team to decide who met our discharge criteria. In our study, 6 patients needed inpatient admission directly from the DSU, and one more patient was readmitted after 24 h due to spreading ecchymosis at the site of surgery. This gives a conversion rate of 9.8%. The various reasons for inpatient admission from the DSU are shown in Table 3.

**Table 3:** Rate of inpatient admission from the DSU

Complications needing inpatient admission	Frequency	Percentage of total
Bleeding and haematoma	3	4.2
Severe pain	1	1.4
More extensive surgery needed	2	2.8
Delayed readmission after 24 h	1	1.4
Total	7	9.8

Other recognized complications in 3 patients (4.2%) did not warrant conversion to inpatient admission. Two cases of broken screws could not be retrieved and a case of incomplete retrieval of the foreign body. These patients were counselled appropriately about the problem encountered and scheduled for repeat procedures but did not show up at the scheduled date.

**Fig. 2:**
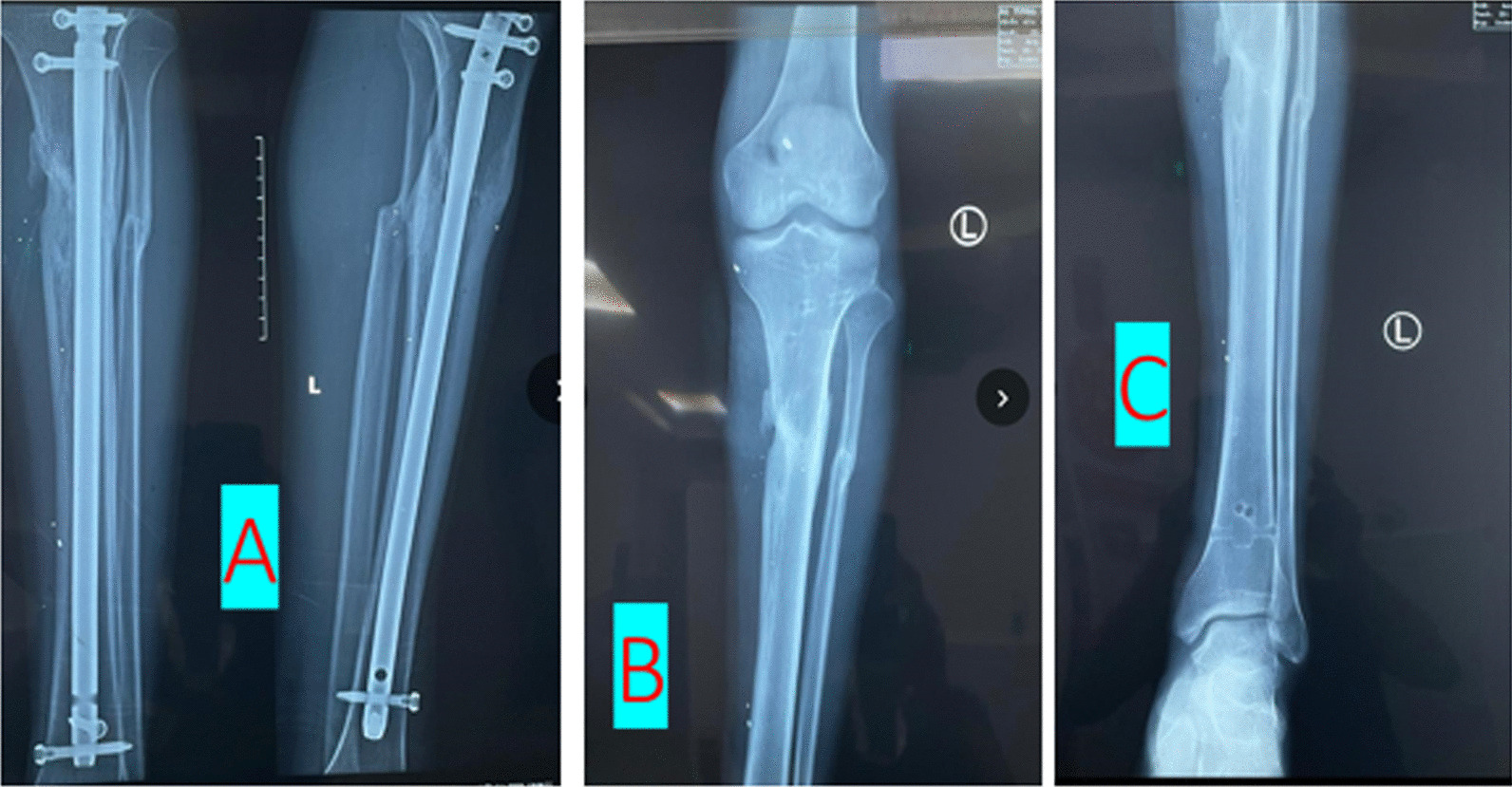
**A** Healed left tibia fracture with IM nails, **B**, **C** Post-removal radiographs

**Fig. 3:**
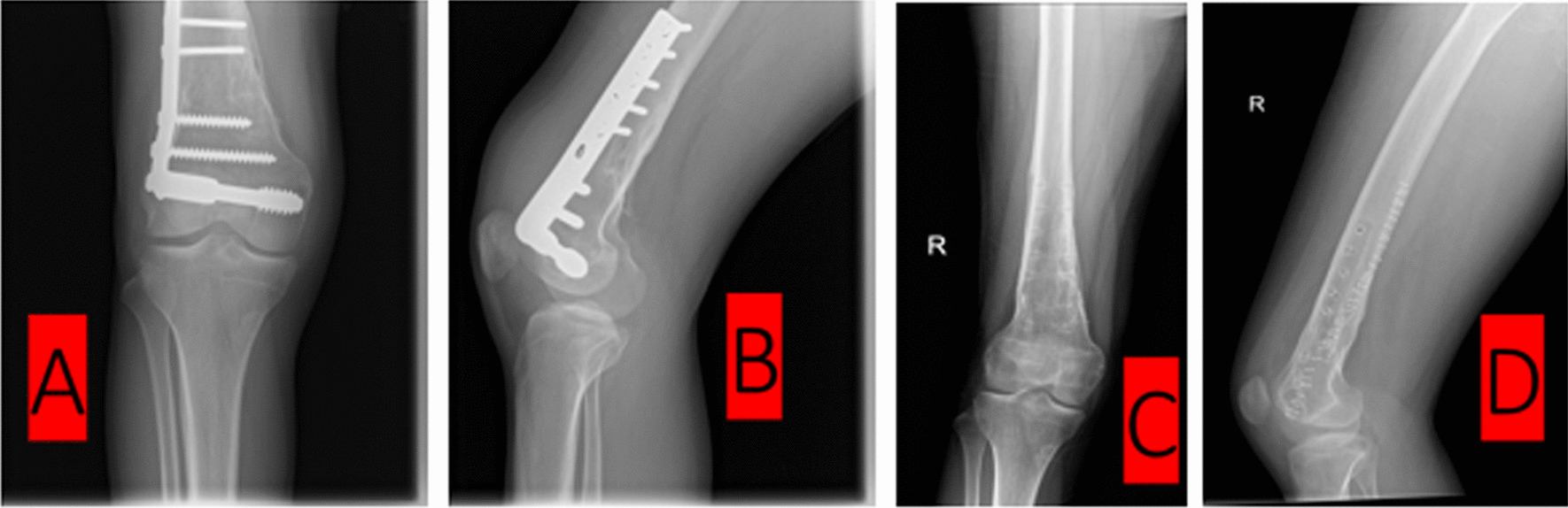
**A**, **B** Healed fracture of the right distal femur, fixed by dynamic condylar screw; **C**, **D** Post-removal radiographs

**Fig. 4:**
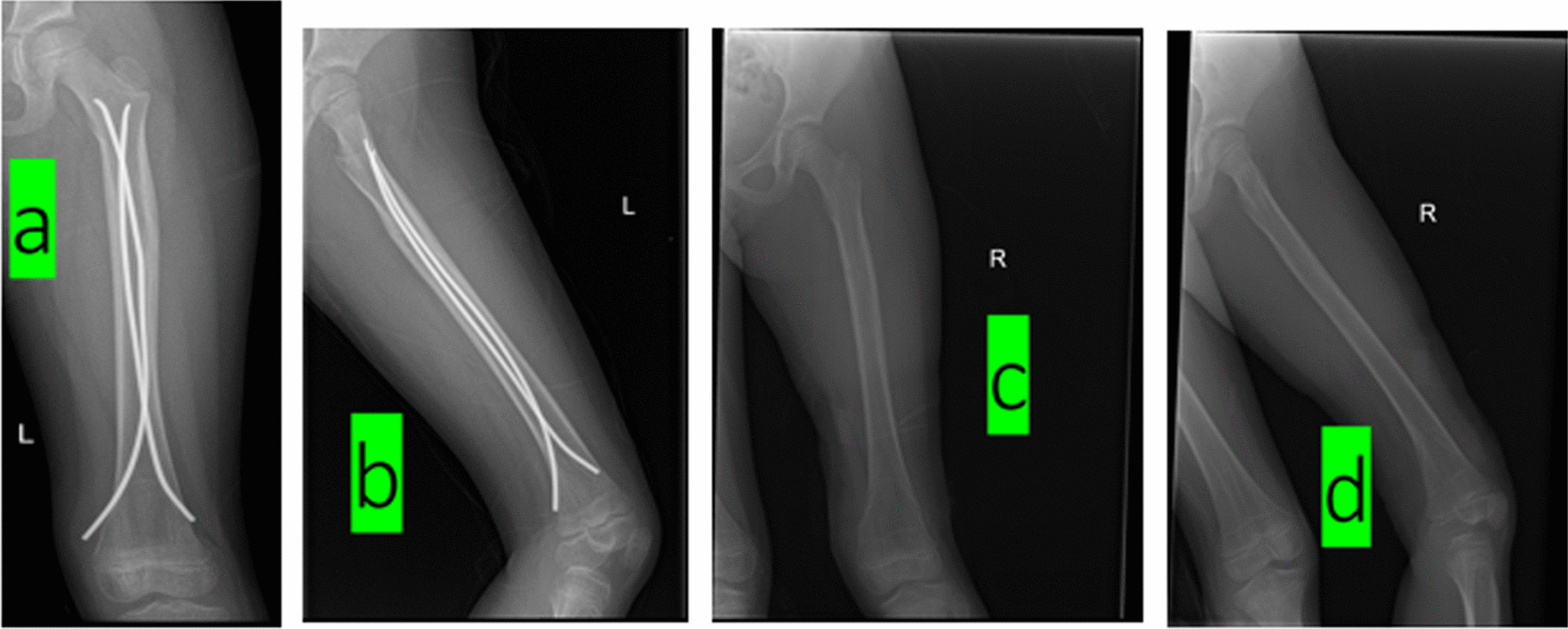
**a**, **b** Healed left femur fracture with elastic nail fixation; **c**, **d** Post-removal radiographs

Some of the cases (radiographs) are shown in Figs. 2, 3 and 4.

## Discussion

The trends and pattern of utilisation of day-case procedures in orthopaedic practice continue to evolve with improvements in anaesthetic and perioperative care. The age distribution in our study was 4 to 55 years, with a mean of 25.3 ± 12.2. This is in slight contrast to other studies that reported a wide age distribution. Malizu et al. [12] reported ages of 8 months to 76 years, Ajibade et al. [13] reported ages of 4 weeks to 78 years, and Mulimba et al. [14] reported ages of 2 to 75 years. Our narrow age range was partly due to patient selection bias by the surgeons and partly because some older patients explicitly rejected day-case procedures and preferred inpatient admission.

During the study period, our day-case rate was 7.8% (71 out of 914 elective orthopaedic procedures). The rate is comparable to that of Malizu et al. [12] (9.8%) and Ajibade et al. [13] (3.48%). However, the Royal College of Surgeons ‘Guidelines for Day-Case Surgery’ suggested a day-case target for elective surgery of 50% [6]. The details of our procedures revealed that 59 cases (83.1%) were removal of metalwork. This is similar to the study by Malizu et al. [12], who reported the removal of implants as the most common day-case procedure, comprising 59.6% of their cases. The spectrum of our day-to-day case procedures was limited compared to other studies with a broader spectrum of cases. A report by John Older [15] working at a district general hospital in the UK reported a broader scope of day-case procedures, including arthroscopic knee surgeries and hallux valgus corrective osteotomies. Similarly, the study by Ajibade et al. [13] in Nigeria and Mulimba et al. [12] in Kenya reported a broader scope and complexity of orthopaedic day-case procedures, including open reduction and internal fixation of fractures, sequestrectomies, amputations, arthroscopies of the knee, tendon and nerve repairs.

In our study, 65 cases (91.5%) were ASA grade I, whilst the remaining 6 cases (8.5%) were ASA grade II. There were no ASA grade III and above, which was mainly due to our selection criteria and the anaesthetist only accepting ASA I and II grades. In the study by Malizu et al. [12], all their cases were ASA grade I. The anaesthetic techniques used in our study were spinal (*n* = 30, 42.3%), general (*n* = 22, 31.0%), local (*n* = 11, 15.5%) and regional (*n* = 8, 11.3%). In a report by Bailey et al. [16] titled ‘Guidelines for day-case surgery 2019’, which represent guidelines from the Association of Anaesthetists and the British Association of Day Surgery, they suggested that fitness for a procedure should relate to the patient’s functional status rather than ASA physical status. However, our hospital DSU policy seems to agree with a report by Chraralambous et al. [17] in which they only considered medically fit (ASA I and II patients) for day-case orthopaedic procedures.

Our study revealed the conversion rate to inpatient admission (unplanned admission) after day-case procedures to be 9.8%; 7 out of 71 cases were admitted for various reasons, as revealed in Table 3. One of our patients was readmitted after 24 h when he called to raise concerns about a spreading ecchymosis at the surgery site. Alsaegh et al. [18], in a retrospective study covering a period of 6 years, reported a conversion rate of 3.4%. Another study by Hardman et al. [19] noted a lower conversion rate of 0.41%, which varied across different surgical specialities, whilst O’Connor et al. [20] recorded an aggregated conversion rate of 2.7% across surgical specialities, with Orthopaedics having the highest conversion rate of 1.07%.

The complication rate in our study was 14.0% in 10 patients, with 7 of them (9.8%) requiring inpatient admission and 3 cases (4.2%) not requiring inpatient admission. Most of the complications were related to surgery and were managed. We did not identify patient-related factors in the development of the complications, and there was no significant morbidity. However, one patient had severe pain, necessitating inpatient admission for pain control in consultation with the anaesthesia unit. The patient had an implant removed from the femur under spinal anaesthesia. In their study, Malizu et al. [12] reported a 30% complication rate, most related to surgery. Their study's complication rate correlated with the procedure's duration, which was not assessed in the current study.

Our dedicated DSU has helped plan procedures with minor morbidity from complications. There was no cancellation of any operation in our study, which may be attributed to the fact that we usually allow patients to reschedule before their given date during the pre-assessment stage.

## Conclusion

Day surgery is well established in our facility, which is a moderately sized district hospital. However, the utilisation of dedicated DSU was low even though patients have a reasonable level of acceptability. Developing a more detailed protocol for day surgery will help optimise patient selection criteria, enhance preoperative assessment, and improve postoperative care pathways. This will, in turn, increase the spectrum and number of procedures performed and enhance patients’ overall experience.

## Data Availability

The datasets used and analyzed during the current study are available from the corresponding author upon reasonable request.

## Abbreviation

DSU: Day surgery unit
